# The International Academy for Clinical Hematology (IACH) 2026–2030 strategic vision and roadmap: Building the global reference for education, collaboration, and innovation in clinical hematology

**DOI:** 10.46989/001c.163209

**Published:** 2026-06-15

**Authors:** Mohamad Mohty, Bipin Savani, Ali Bazarbachi, Didier Blaise, He Huang, Florent Malard, Mohamed Kharfan-Dabaja, Thierry Facon, Eolia Brissot, Zina Peric, Annalisa Ruggeri, Amer M. Zeidan, Naveen Pemmaraju, Zeev Shoham, Junia V. Melo, Arnon Nagler

**Affiliations:** 1 Sorbonne Université; Centre de Recherche Saint-Antoine, INSERM UMRs 938; Service d’Hématologie Clinique et de Thérapie Cellulaire, Hôpital Saint Antoine, AP-HP, Paris, France; 2 Vanderbilt University Medical Center, Nashville, Tennessee, USA; 3 Transplantation & Cellular Therapy, Department of Hematology, Institut Paoli Calmettes, Management Sport Cancer Lab, Luminy, Aix Marseille University, Marseille, France; 4 Bone Marrow Transplantation Center, the First Affiliated Hospital, Zhejiang University School of Medicine, and the Liangzhu Laboratory, Zhejiang University School of Medicine, Hangzhou, China; 5 Division of Hematology and Medical Oncology, Bone Marrow Transplantation and Cellular Therapy Program, Mayo Clinic, Jacksonville, Florida, USA; 6 University of Lille, CHU de Lille, Service des Maladies du Sang, Lille, France; 7 Faculty of Medicine, University of Rijeka and University Hospital Centre Rijeka, Rijeka, Croatia; 8 Hematology and BMT unit, IRCCS San Raffaele Hospital, Milano, Italy; 9 Section of Medical Oncology and Hematology, Department of Internal Medicine, Yale School of Medicine, and Yale Cancer Center, New Haven, CT, USA; 10 Department of Leukemia, The University of Texas MD Anderson Cancer Center, Houston, TX, USA; 11 Hadassah Medical School, Affiliated to the Hebrew University, Ein Kerem, Jerusalem, Israel; 12 Faculty of Health and Medical Sciences, University of Adelaide, SA 5000, Australia; 13 Division of Hematology, Sheba Medical Center, Tel Hashomer, Israel

**Keywords:** IACH, Education, Roadmap, Collaboration, Innovation

## Abstract

The International Academy for Clinical Hematology (IACH) is a global, non-profit educational organization dedicated to advancing excellence, equity, and innovation in clinical hematology. Since its creation, the IACH has built an international community spanning more than 130 countries, leveraging digital-first education, scientific collaboration, and professional networking to improve patient care worldwide. This document presents the IACH Strategic Vision for 2026–2030, outlining a structured roadmap for the Academy’s next phase of growth and international leadership. The strategy is built upon six interdependent pillars: scientific excellence and credibility; digital leadership and intelligent learning; academic alliances and global footprint; innovation, visibility, and advocacy; transparency and sustainability; and brand positioning with global impact. By reinforcing scientific rigor, embracing digital transformation, and promoting innovation, the IACH aims to become the most trusted global reference for clinical hematology education and collaboration. This strategic vision positions the IACH as a unifying platform to unite the world hematology community under one banner of knowledge, collaboration, and compassion, for the benefit of every patient, everywhere.

## INTRODUCTION

The International Academy for Clinical Hematology (IACH) is a non-profit educational organization devoted to advancing excellence in clinical hematology worldwide. It was founded in 2018 by an international group of physicians focused on promoting good clinical practice. Since its establishment, the IACH has become a prominent platform for knowledge dissemination, professional networking, and continuing medical education, engaging thousands of physicians, scientists, and allied health professionals across more than 130 countries. For the past 7 years, the Academy has rapidly expanded its impact through digital innovation and global collaboration. As of 2025, the IACH operates several specialized disease academies (Chronic Lymphocytic Leukemia, Multiple Myeloma, and Stem Cell Transplantation), and has pioneered multi-stakeholder programs for physicians, nurses, pharmacists, and patients. In a historic milestone, IACH became the first hematology organization worldwide to deliver a complete Artificial Intelligence (AI)-generated educational webinar in 2025. Accredited as an official provider of Continuing Medical Education-Continuing Professional Development (CME-CPD) by the European Board for Accreditation in Hematology (EBAH), the IACH delivers high-quality, independent virtual and in-person programs. Moreover, the IACH publishes an open-access journal, *Clinical Hematology International,* and maintains a comprehensive digital library including podcast series, multilingual content (English, Spanish, Chinese), and a WhatsApp channel for rapid knowledge dissemination.

**Figure 1.**
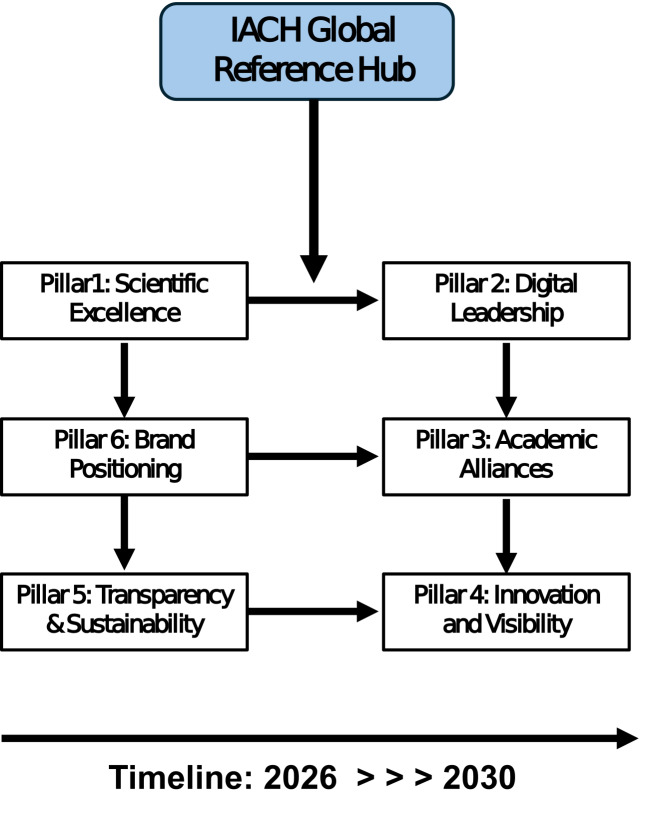
This figure illustrates the six interdependent pillars of the IACH’s strategic vision, highlighting their synergies and collective contribution to the Academy’s mission. The central hub represents the unified goal of becoming the global reference for education, collaboration, and innovation, with arrows demonstrating key interconnections (e.g., scientific credibility underpinning digital learning and academic alliances fostering innovation).

Through a combination of digital innovation, and a collaborative spirit, the IACH has succeeded in uniting a diverse global community of hematologists under a shared commitment: to improve patient care and foster equity of access to cutting-edge education and research. This document presents the strategic vision for 2026-2030, articulating the next stage in the IACH evolution. It aims to define clear objectives, priorities, and implementation frameworks designed to position the IACH as the global reference institution for clinical hematology education, consensus building, and digital collaboration. The strategy rests on six interdependent pillars: (i) scientific excellence and credibility, (ii) digital leadership and intelligent learning, (iii) academic alliances and global footprint, (iv) innovation, visibility, and advocacy, (v) transparency, and sustainability, and (vi) brand positioning and global impact. To inform this strategic vision, a SWOT (S – Strengths; W – Weaknesses; O – Opportunities; and T – Threats) analysis highlighted the IACH’s current position: strengths include its digital-first approach, global community in over 130 countries, EBAH-accredited CME programs, and the indexed journal *Clinical Hematology International*. Weaknesses encompass potential digital access barriers in low- and middle-income countries and reliance on limited funding sources. Opportunities lie in AI-driven innovations, expanding regional chapters, and expanding initiatives for a wider audience of stakeholders. Threats involve competition from non-academic entities and economic pressures on non-profit funding. This analysis underscores the need for the six pillars to leverage strengths and address gaps. Each pillar is elaborated as a strategic axis with rationale, actions, and measurable outcomes (**Figure 1**).

## SCIENTIFIC EXCELLENCE AND CREDIBILITY

Scientific integrity is the foundation of the IACH’s legitimacy and impact. In a rapidly evolving therapeutic landscape, hematology requires platforms that provide independent interpretation of evidence, harmonized recommendations, and translational continuity between research and practice. Between 2026 and 2030, the IACH will strengthen its scientific authority through different complementary initiatives, including the establishment of the IACH consensus and practice frameworks series, and the development of a dedicated IACH clinical research network (IACH-CR).

The consensus frameworks series will aim to deliver updated, peer-reviewed position statements on major disease entities and cross-cutting themes and topics. These frameworks will involve international experts from allied societies and follow a transparent process to ensure representativeness and reproducibility. On the other hand, the IACH-CR network will serve as a collaborative, non-commercial data platform, enabling real-world evidence generation in specific domains. The IACH-CR network will adhere to the highest standards in terms of data protection, security protocols, anonymization techniques, and regular audits.

In addition to its PubMed-indexed journal *Clinical Hematology International*, by 2030, the IACH aims to be recognized as a trusted source of clinical guidance for operational clinical research activities across multiple centers.

## DIGITAL LEADERSHIP AND INTELLIGENT LEARNING

The IACH was founded as a digital-first institution. The post-COVID pandemic era has confirmed that sustainable education must be global, interactive, and electronic data-driven. Digital leadership will therefore remain a central pillar of the Academy’s mission. As such, the IACH will invest in the creation of a next-generation digital ecosystem, the IACH “Digital Campus 2.0”, incorporating adaptive learning technology, AI-assisted curation, and integrated CME/CPD certification. The Digital Campus 2.0 will offer modular courses structured by disease area, with progressive certification levels. Completion will grant CME credits stored on a secure blockchain to ensure traceability and international recognition. A comprehensive knowledge hub will integrate live webinars, the on-demand educational library, interactive sessions, and AI-generated digests of recent scientific literature. Personalized dashboards will track user engagement and knowledge acquisition. The IACH will establish an internal learning analytics unit responsible for monitoring participation metrics, satisfaction rates, and practice-impact indicators. Findings will be summarized in an annual education impact report, positioning the IACH as a leader in evidence-based medical education. By 2030, the platform will serve thousands active global users, host hundreds of CME-accredited modules, and produce yearly analytics reports disseminated to the hematology community, sponsors, and regulatory bodies.

## ACADEMIC ALLIANCES AND GLOBAL FOOTPRINT

To sustain long-term scientific and educational excellence, the IACH must anchor its activities within academic institutions and expand its geographical reach. Collaborative partnerships ensure mutual credibility, resource sharing, and cultural diversity. The IACH will establish structured alliances with major centers of excellence and develop regional chapters to ensure local ownership of the IACH vision. From 2026 onward, the IACH will formalize memoranda of understanding with several leading academic centers across Europe, the Americas, the Middle East, Asia, and Africa. Each partner institution will have the possibility to host IACH-supported workshops, and events combining local case discussions, mentoring, and digital outreach. Also, regional chapters will be progressively launched (eg. IACH-China, IACH-USA, IACH-LATAM, IACH-MENA, etc., each guided by local experts. This federated model will ensure contextual relevance while maintaining the unified IACH identity. Finally, an IACH global fellowship and mentorship program will be established to support early-career hematologists. Several fellows per year will be paired up with senior mentors for hybrid programs, including webinars, collaborative projects, and travel grants to IACH events. By 2030, the IACH aims to operate through different active regional chapters, maintain partnerships with academic institutions, and have trained dozens of fellows. The resulting academic network will constitute a new global ecosystem of shared expertise.

## INNOVATION, VISIBILITY, AND ADVOCACY

To achieve durable influence, the IACH must not only educate but also shape discourse, connect stakeholders, and anticipate the next frontiers of hematology. The Academy will institutionalize innovation through its different meetings, a system of awards recognizing excellence, and a professional communication strategy to enhance visibility and advocacy. Beginning in 2026, the IACH will convene intense discussions with academic leaders, regulatory authorities, industry partners, and patient representatives to discuss transformative topics such as AI , cell therapy access, and sustainability in healthcare.

The IACH plays a unique and powerful advocacy role for rare blood cancers (e.g. Blastic Plasmacytoid Dendritic Cell Neoplasm, BPDCN ), by uniting experts, amplifying research, and giving visibility to fields that are too often overlooked. By building global networks and dedicated platforms, the academy helps researchers connect, collaborate, and raise awareness, ultimately accelerating progress for patients who need it most.

The IACH awards program will recognize outstanding contributions across categories (e.g. Lifetime Achievement, Emerging Leader, Patient Advocacy, etc.) presented during the IACH Annual Meeting. Regular press releases, expert interviews, and short video features will disseminate the Academy’s achievements to both professional and public audiences. With such strategy, we expect the IACH to become a visible thought leader, producing globally cited reports, and achieving regular coverage in high-impact outlets.

## TRANSPARENCY AND SUSTAINABILITY

Integrity and sustainability are essential to maintaining independence and trust. The IACH’s governance model should always balance scientific freedom with operational accountability. Thus, a multi-sponsor educational framework should be the preferred option. Each disease area will be supported by several industry partners under strict conflict-of-interest policies. The IACH Foundation will administer fellowships, awards, and philanthropic initiatives. By 2030, the IACH will sustain a balanced financial portfolio, with a preference funding derived from non-pharmaceutical sources.

## BRAND POSITIONING AND GLOBAL IMPACT

Visibility and perception define long-term institutional value. The IACH brand must reflect scientific authority, accessibility, and independence while projecting a unified global identity. It will consolidate its public image through consistent messaging, impact reporting, and high-level partnerships with leading international organizations. A unified brand platform will articulate the IACH’s guiding statement: “Hematology Without Borders.” This mantra builds on the IACH’s established ethos, in promoting diversity, equity, inclusion, innovation, inspiration, open and collaborative mindset and, most importantly, improving patients’ outcome, because patients matter to us wherever they live.

All digital and printed materials will follow standardized design and communication guidelines to reinforce recognition. The Academy will publish an annual report, quantifying participation, reach, citation impact, and educational outcomes. Collaborations with sister-societies, and global patient organizations will be pursued to align advocacy and capacity-building efforts. The goal is to allow the IACH to be universally acknowledged as one of the most influential global hematology education organizations, with demonstrable impact on practice and policy.

## CONCLUSION

The IACH strategic vision for 2026–2030 represents a decisive step toward institutional maturity and international leadership. By coupling scientific rigor with innovation and inclusivity, the IACH will consolidate its role as a bridge between continents, generations, and disciplines in the service of patients with hematologic disorders. While ambitious, this vision acknowledges potential implementation risks, including technological adoption barriers in resource-limited regions, funding volatility, and evolving regulatory landscapes for data and AI. Mitigation strategies will include phased rollouts with pilot testing, diversified revenue streams beyond industry sponsors, inclusive design for low-bandwidth access, and ongoing compliance with global standards. Regular stakeholder feedback and adaptive planning will ensure resilience, aligning with the IACH’s commitment to agility and “maverick thinking” for sustained progress.

The IACH will remain guided by its founding values, independence, excellence, and solidarity, while embracing a future defined by digital transformation and collaborative discovery. Our 2030 vision is to unite the international hematology community under one banner of knowledge, collaboration, and compassion, for the benefit of every patient, everywhere.

### STATEMENTS AND DECLARATIONS

The authors declare no competing financial interests in relation with this work.

### ETHICAL APPROVAL

Not applicable.

### CONSENT TO PARTICIPATE/INFORMED CONSENT

Not applicable.

### CONSENT FOR PUBLICATION

Not applicable.

### AUTHOR CONTRIBUTION - CREDIT

Writing – original draft: Mohamad Mohty, Bipin Savani, Ali Bazarbachi, and Arnon Nagler; Writing – review & editing: All the authors.

### CONFLICT OF INTEREST AND EDITORIAL INDEPENDENCE STATEMENT

Mohamad Mohty, Bipin Savani, Ali Bazarbachi, and Arnon Nagler are editorial board members of *Clinical Hematology International* and are co-authors of this manuscript. To ensure editorial independence and avoid any potential conflict of interest, they had no role in the selection of reviewers, peer-review evaluation, editorial discussions, or the final publication decision. The manuscript was handled entirely by an independent editor, in accordance with the journal’s standard peer-review procedures.

